# Analysis of *MTR* and *MTRR* Polymorphisms for Neural Tube Defects Risk Association

**DOI:** 10.1097/MD.0000000000001367

**Published:** 2015-09-04

**Authors:** Yongxin Wang, Yuan Liu, Wenyu Ji, Hu Qin, Hao Wu, Danshu Xu, Turtuohut Tukebai, Zengliang Wang

**Affiliations:** From the Neurosurgical Department, the 1st Affiliated Hospital of Xinjiang Medical University, Urumqi, China.

## Abstract

Neural tube defects (NTDs) are the most common congenital defects of the central nervous system among neonates and the folate status during pregnancy was considered as the most important etiopathogenesis of NTDs. Besides, methionine synthase (MTR) gene and methionine synthase reductase (MTRR) gene were folate metabolism involved genes and had been investigated in several previous studies with inconsistent results. Hence, we aimed to explore the association of 4 selected single-nucleotide polymorphisms (SNPs) on MTRR/MTR gene and the susceptibility of NTDs in a Chinese population.

Seven SNPs were selected from HapMap databases with Haploview 4.2 software. A polymerase chain reaction-restriction fragment length polymorphism (PCR-RFLP) was performed to genotype the polymorphisms from blood samples of 165 NTDs patients and 280 healthy controls. The correlation between these SNPs and NTDs risk was tested by Student *t* test and Chi-square test by STATA 11.0 software. Furthermore, we performed a meta-analysis of relevant studies to investigate the association between the SNPs *MTRR* 66A>G and *MTR* 2756A>G and the susceptibility of NTDs.

An increased risk of NTDs was verified to be significantly associated with *MTRR* 66A>G (G allele vs. A allele: OR = 1.36 (1.03–1.80), *P* = 0.028; GG + AG vs. AA: OR = 1.60 (1.05–2.43), *P* = 0.027) and *MTR* 2756A>G (G allele vs. A allele: OR = 1.45 (1.06–1.98), *P* = 0.021; GG + AG vs. AA: OR = 1.51 (1.02–2.23), *P* = 0.038) in our study. However, the other SNPs in our analysis showed no significant association with NTDs risk (all *P* > 0.05). Furthermore, the result of the meta-analysis supported the association between MTRR 66A>G and NTDs risk (G allele vs. A allele: OR = 1.32, 95% CI = 1.09–1.61, GG + GA vs. AA: OR = 1.49, 95% CI = 1.06–2.09, GG vs. AA: OR = 1.61, 95% CI = 1.04–2.49).

Our study confirmed that the *MTRR* 66A>G and *MTR* 2756A>G were significantly associated with the increased NTDs risk in a Chinese population. The further meta-analysis enhance that *MTRR* 66A>G was connected with the susceptibility of NTDs widely. Further investigations based on more detailed stratification were recommended.

## INTRODUCTION

Neural tube defects (NTDs) are the most common and complicated congenital defects of the central nervous system at birth.^1,2^ The representations of NTDs range among various phenotypes including spina bifida, anencephaly, and encephalocele. Moreover, NTDs occurred around 1 to 28 days after conception as reported in previous studies.^3,4^ According to the epidemiology, the incidence of NTD's affection is about 0.2% worldwide. Merely, the rate of NTDs ranges among races and areas in previous studies. It was also observed that NTDs reach up to 25% of all the birth defects.^5^ Furthermore, NTDs were acquired or inherited with multifactorial pattern under both environmental and genetic influential factors during pregnancy. Low level of folate, vitamin B12 and high level of homocysteine in the serum during pregnancy were all established to increase the risk of NTDs.^6^ And it was confirmed in earlier researches that proper ingestion of folate acid could decrease the occurrence and recurrence of NTDs notably.^7–9^ Besides, various investigations paid attention to the association between the single nucleotide polymorphisms (SNPs) on folate-related genes and risk of NTDs. Consequently, elucidation of the NTDs mechanisms and focusing on candidate genes that are involved in folate-related pathways could be informative for developing improved prevention strategies and reducing the global burden of NTDs.^10^

The gene of methionine synthase (*MTR*) and methionine synthase reductase (*MTRR*) were 2 genes involved in the folate metabolism and had been investigated in several previous studies. It had been reported that the polymorphisms on the gene of *MTR* and *MTRR* acted as risk factors for NTDs. *MTR* gene could encodes the enzyme of vitamin B_12_-dependent and further converts 5-methyltetrahydrofolate (5-methyl THF) and homocysteine to tetrahydrofolate (THF) and methionine, respectively.^11^ This process is essential for maintaining ample tetrahydrofolate and methionine pools in the cell. On the other hand, *MTRR* gene is one of the most important genes and has been studied deeply among all the folate metabolism related genes. *MTRR* functions as an activation partner of *MTR* which could contribute to the demethylation of homocysteine (Hcy) to methionine through cobalamin and subsequently regenerates *MTR*.^12^ At the 66 bp of *MTRR* gene, an A to G switch (66A>G) has been widely studied and verified to result in the deficiency of *MTRR* in patients via the conversion of isoleucine to methionine (I22M).^13,14^ On the contrary, the defect of *MTRR* could subsequently affect the function of *MTR* and disrupt the methionine/homocysteine cycle.

In our study, we aim at 4 nonsynonymous polymorphisms (2756A>G (rs1805087), −1003A>G (rs10925250), +370G>C (rs12060570) and −1059T>C (rs10925235)) within the gene of *MTR* and 3 potential SNPs (66A>G (rs1801394), 1049A>G (rs162036), and 524C>T (rs1532268)) on the *MTRR* gene to investigate their association with the NTDs risk. All the SNPs were identified from public databases and tended to be representatives of the SNPs. However, considering the limitation of individual study with small sample size and the significant controversy of relevant studies, a further meta-analysis with public data for the purpose of conclude an exhaustive conclusion of empirical evidence to prove the association between the NTDs risk and the SNPs of *MTRR* 66A>G and *MTR* 2756A>G was also performed.

## MATERIALS AND METHODS

### Ethnic Statement

Our study was conducted in strict accordance with the protocol approved by the Ethics Committee and parental informed consent of the 1st Affiliated Hospital of Xinjiang Medical University. An informed consent granted by this ethics committee must be signed by each volunteer (as for the neonates, the informed consent must be signed instead by their parents or guardians).

### Study Population

A total of 445 neonates in Chinese Han population were enrolled from August 2013 to December 2014. The subjects were consisted of 165 confirmatory NTDs patients from the Department of Pediatric Neurology who were identified using routine ultrasound scanning and 280 healthy controls who were randomly selected from the unrelated healthy neonates from inpatients of the gynecology and obstetrics department as outlined in Table 1. We collected 5 mL venous blood sample with EDTA vacutainer from each subject after their guardians provided a written informed consent. The clinical characteristics of all the cases and controls were collected as well. Further, regarding to the influencing factors which may related to NTDs of the neonates. The levels of serum cobalamin, serum folate, RBC (red blood cell) folate, apo-haptocorrin, and apo-transcobalamin were all quantified with the reagents and system of Ciba (Ciba Corning Diagnostics Corp., Medfield, MA). Similarly, the level of total homocysteine (tHcy) in plasma was detected with high-pressure liquid chromatography. All the samples and complete follow-up data were available for each specimen (more clinical characteristics are present in Table 1).

**TABLE 1:**
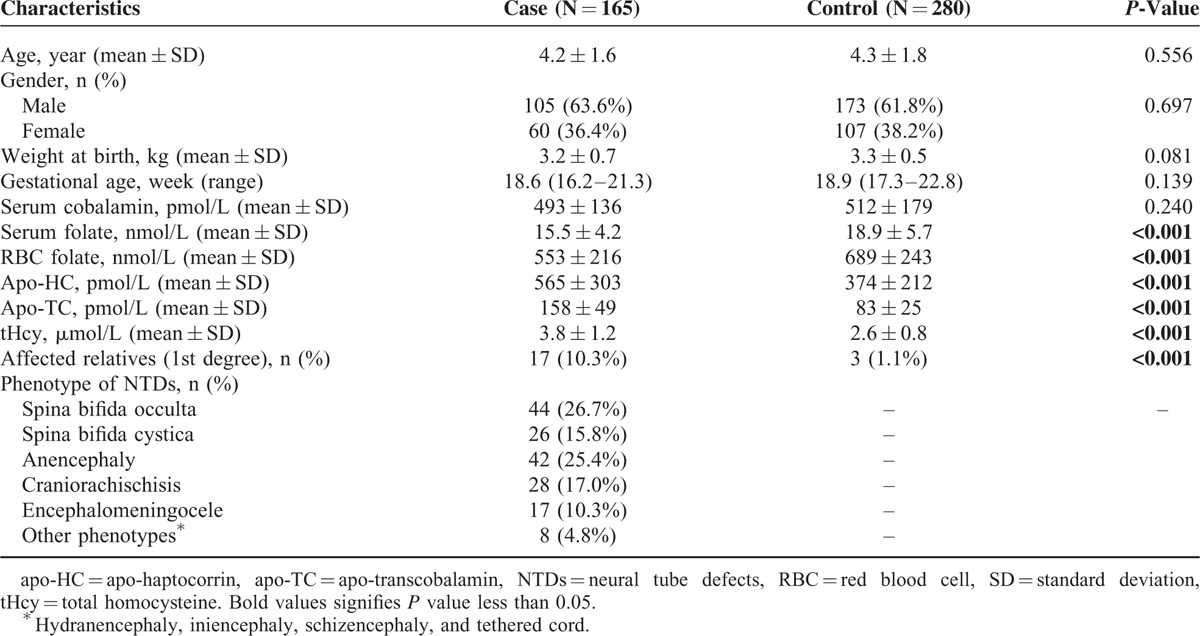
Characteristics of Cases and Control Children

### SNPs Selection

**FIGURE 1:**
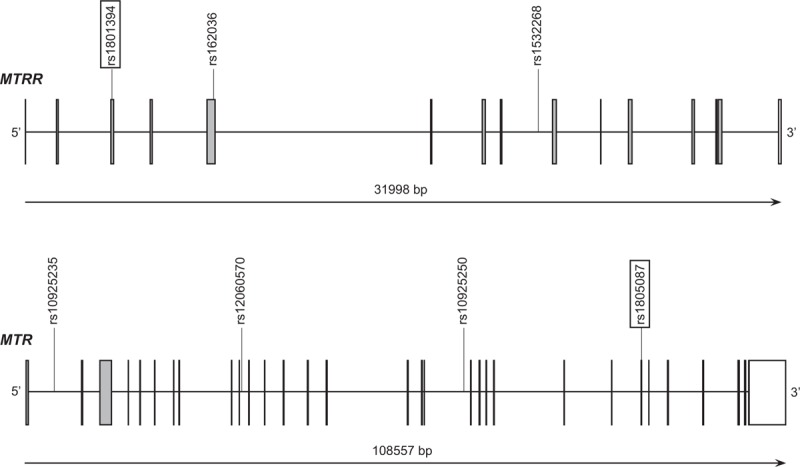
Locations of the selected SNPs. On the gene of *MTR*, SNP 2756A>G was located at exon 25, −1003A>G was located at intron 18, +370G>C was located at intron 10 and −1059T>C was located at intron 1. On the gene of *MTRR*, SNP 66A>G, 1049A>G, and 524C>T were located on exon 1, intron 5, and intron 8 regions, respectively.

The polymorphisms in our study were retrieved from online HapMap database (HapMap Data Rel 24/phaseII Nov08, on NCBI B36 assembly, dbSNP b126) of Chinese HapMap Consortium. And critical polymorphisms were identified with Haploview 4.2 (Broad Institute, Cambridge, MA) software, with the linkage disequilibrium (LD) pattern under HapMap genotype data and r^2^ of 0.8 was selected as a threshold for the analyses.^15^ As a result, we select 3 SNPs as representatives on *MTRR* gene (66A>G, 1049A>G, and 524C>T) and 4 SNPs (2756A>G, −1003A>G, +370G>C and −1059T>C) on *MTR* gene via our filter. Additionally, the SNP 66A>G on *MTRR* gene and SNP 2756A>G on *MTR* gene had been reported with high incidence and were supposed to result in amino acid change which would further vary the function of folate relevant enzymes.^16^ The accurate locations of the selected SNPs were as following. On the gene of *MTR*, SNP 2756A>G was located at exon 25, −1003A>G was located at intron 18, +370G>C was located at intron 10 and 1059T>C was located at intron 1. On the gene of *MTRR*, SNP 66A>G, 1049A>G, and 524C>T were located on exon 1, intron 5, and intron 8 regions, respectively, as demonstrated in Figure 1.

### Genotyping Method

DNA was extracted from the whole blood of both NTD cases and controls by QIAamp DNA Blood Mini Kit (Qiagen, UK) using proteinase K and RNase A, isopropanol, cold acetic acid, and stored at −20°C. Further, the genotyping of selected SNPs were done by polymerase chain reaction-restriction fragment length polymorphism (PCR-RFLP) analysis. And the quality of genotyping was tested by direct sequencing with >99% agreement for the 5 polymorphisms or repeat analysis genotyping of at least 10% of the samples by the initial genotyping method.^17,18^ The SNPs primers we used for PCR amplification were designed with Primer Premier 5 software as shown in Table 2 with their related restriction enzymes and reaction condition. The primers and conditions of all assays are available upon request.

**TABLE 2:**
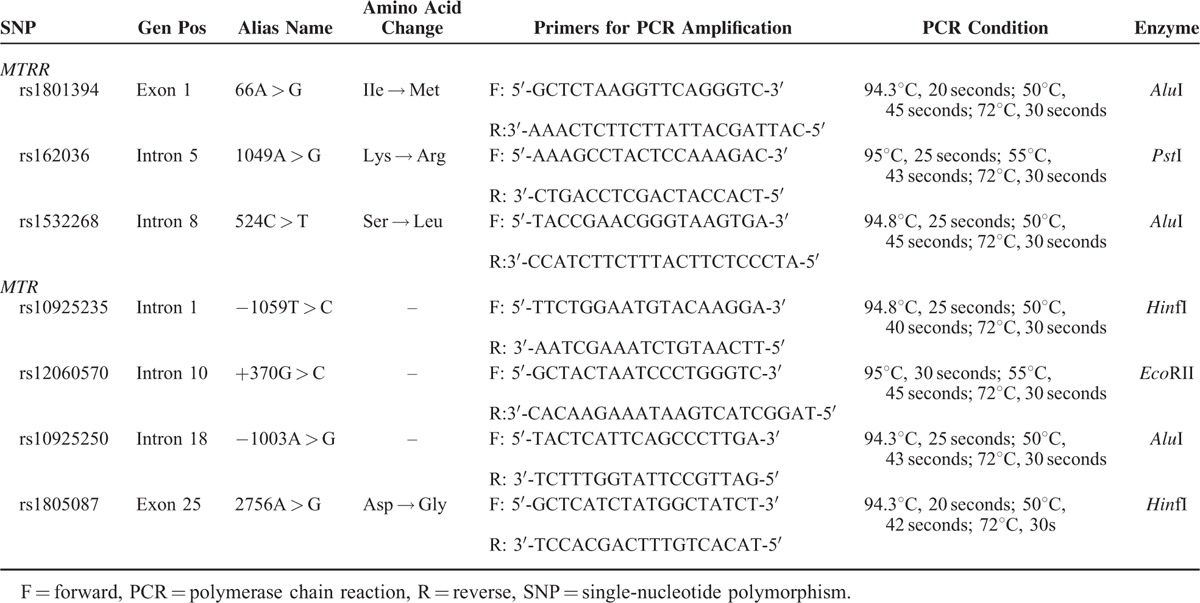
Primers of *MTR* and *MTRR* Genetic Polymorphisms for PCR Amplification

### Statistical Analysis

We performed all the statistical analysis in our case–control study with the STATA version 11.0 software (StataCorp LP, Texas 77845-4512 USA). During our analysis, the difference of clinical characteristics and frequency of genotypes between NTDs patients and controls was compared by Student *t* test and Pearson Chi-square (χ^2^) test. We also performed a chi-square goodness-of-fit test by comparing the observed and expected genotype frequencies to verify the data quality in each subject class, exploring significant departure from Hardy–Weinberg equilibrium (HWE) and investigate the representativeness of sample population. And the genotypes related NTDs risk was evaluated by odds ratios (ORs) with their corresponding respective confidence intervals 95% (CIs) value, for both combined and respective genotype. A 2-sided *P*-value less than 0.05 was considered to indicate statistical significance for all analyses. Further, we conducted a meta-analysis of all relevant studies on the interaction between folate relevant SNP *MTRR* 66A>G and *MTR* 2756A>G and the NTDs risk.

## RESULTS

### Study Characteristics

We enrolled 445 neonates as blood sample donate subjects in present study. The subjects were comprised of 165 NTDs (male = 105, female = 60) patients with the average age of 4.2 (SD = 1.6) and 280 healthy controls (male = 173, female = 107) with the average age of 1.3 (SD = 1.8). Despite the difference among the gender and ages, no significant difference was observed (*P* = 0.697 and *P* = 0.556, respectively). Similarly, the weight at birth of the neonates was also matched between the 2 groups (*P* = 0.081). Further, the tHcy and nutrient levels of each subject were tested. Cobalamin and folate levels were significantly lower and apo-haptocorrin (apo-HC), apo-transcobalamin (apo-TC), and total homocysteine (tHcy) significantly higher in cases as compared with controls (all *P* < 0.001). However, the difference on the level of serum cobalamin between cases and controls was not significant. Statistically significant difference of first-degree relatives affected by NTDs was also observed between the case and control groups Seventeen individuals in the case group, but only 3 in the control group, had a first-degree relative with NTDs (*P* < 0.001). Phenotypes of NTDs patients were classified intro 6 groups, with spina bifida occulta (26.7%) and anencephaly (17.0%) accounting for the most cases as shown in Table 1.

### Association Between *MTRR*, *MTR* Polymorphisms, and NTDs Risk

As present in Table 3, among 3 genotyped SNPs in *MTRR* gene in the present study, only *MTRR* 66A>G was found to be significantly associated with an increased risk of NTDs (G allele vs. A allele: OR = 1.36 (1.03–1.80), *P* = 0.028; GG + AG vs. AA: OR = 1.60 (1.05–2.43), *P* = 0.027). Similarly, for *MTR* gene, only *MTR* 2756A>G was suggested to be linked with the susceptibility of NTDs (G allele vs. A allele: OR = 1.45 (1.06–1.98), *P* = 0.021; GG + AG vs. AA: OR = 1.51 (1.02–2.23), *P* = 0.038). No statistical significant difference was observed between NTDs and other polymorphisms in the present study with all their *P*-value above 0.05, respectively. All these data of result are revealed in Table 3.

**TABLE 3:**
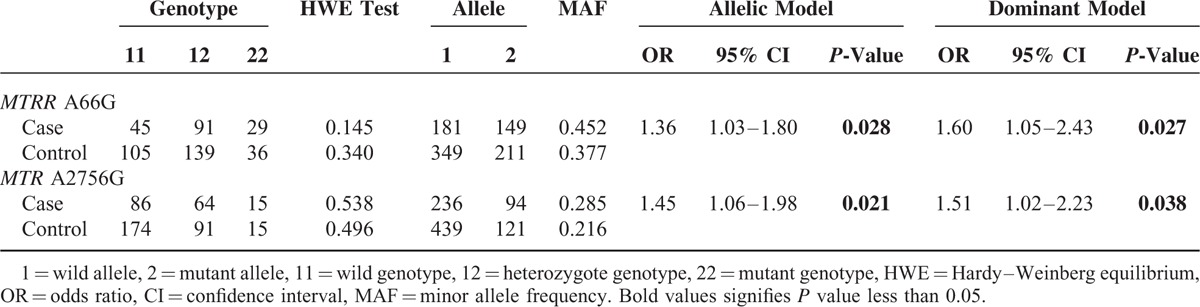
Association Between the *MTR* and *MTRR* Polymorphisms and NTDs Risk

### Meta-Analysis

**FIGURE 2:**
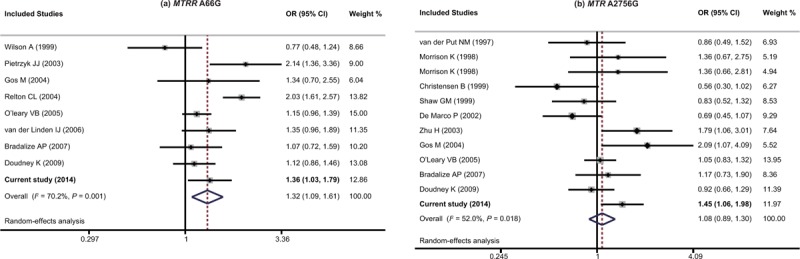
The forest plots of *MTRR* 66A>G and *MTR* 2756A>G in allelic model. (A) *MTRR* 66A>G, (B) *MTR* 2756A>G.

We further performed a meta-analysis to assess the association between the SNPs of *MTRR* 66A>G and *MTR* 2756A>G and the NTDs risk. Fifteen articles were collected with our search strategies in this meta-analysis (including the present study) in total. Among these researches, the data of 5306 specimens were included. Among all the specimens, 3823 were in case of *MTRR* 66A>G (consisted of 1407 cases and 2416 controls) and 3809 were *MTR* 2756A>G (consisted of 1492 cases and 2317 controls). The detailed result of the meta-analysis is revealed in Table 4. The results supported the conclusion of *MTRR* 66A>G as a risk factor for NTDs with *P*-value below 0.05 in allelic model (as shown in Figure 2), dominant model and homozygous model in the meta-analysis with present study (G allele vs. A allele: OR = 1.32, 95% CI = 1.09–1.61, GG + GA vs. AA: OR = 1.49, 95% CI = 1.06–2.09, GG vs. AA + AG: OR = 1.58, 95% CI = 0.85–2.61, GG vs. AA: OR = 1.61, 95% CI = 1.04–2.49). However, overall analysis, regardless with or without the present study, did not give support to the inference that *MTR* 2756A>G might be associated with susceptibility of NTDs (G allele vs. A allele: OR = 1.08, 95% CI = 0.89–1.30, GG + GA vs. AA: OR = 1.15, 95% CI = 0.91–1.45, GG vs. AA + AG: OR = 0.97, 95%CI = 0.67–1.40, GG vs. AA: OR = 1.05, 95% CI = 0.58–1.35).

**TABLE 4:**
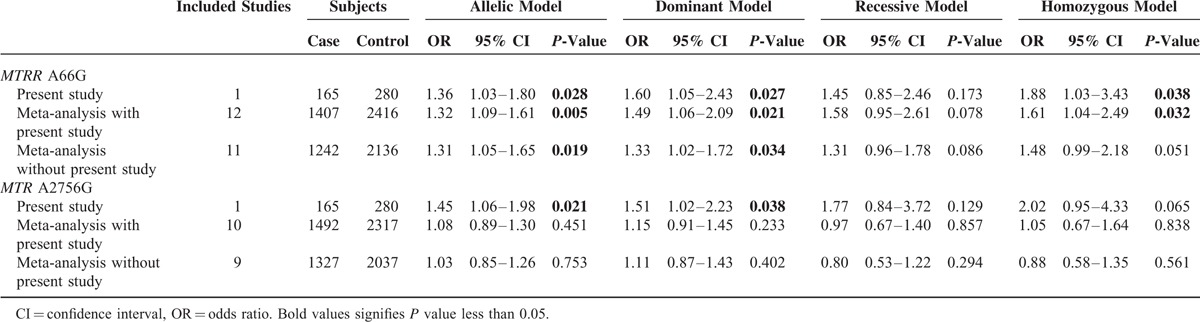
Meta-Analysis of the Association Between the *MTRR* and *MTR* Polymorphisms and NTDs Risk

## DISCUSSION

In the present study, we aim to investigate the association between the risk of NTDs and polymorphisms on the genes of *MTR* and *MTRR*. We revealed that the *MTRR* 66A>G and *MTR* 2756A>G were both significantly associate with an increased NTDs susceptibility. However, the other SNPs we tested in the present study showed no significant associations with the risk of NTDs. This result was subsequently confirmed by a meta-analysis of *MTRR* 66A>G and *MTR* 2756A>G which clarified the association of *MTRR* 66A>G with an increased risk of NTDs.

NTDs were among the most common birth defects widely which could be influenced by the metabolism of folic acid and the level of homocysteine.^19,20^ Various genetic and environmental factors could affect the metabolism of folic acid and further lead to NTDs. Various stratified environmental factors which were related to NTDs have been studied currently.^21^ Accumulating data implicated that the pregnant women without adequate intake of folate from dietary supplements and food were more likely to have a neonate with NTDs.^22^ Consequently, the susceptibility of NTDs could be remedied by proper ingestion of folate supplementation. Additionally, the ingestion of folic acid by 0.4 to 0.6 mg/day was considered as the most favorable quantum.^23^ Furthermore, the persistent low level of folate was also observed in neonates. Besides, according to the latest researches, methionine cycle and folate cycle both have influence on the pathopoiesis of NTDs genetically. In the present study, the folate status was significantly lower in the NTDs neonates. On the contrary, their levels of apo-HC, apo-TC, and tHcy were higher than the healthy controls notably. These implied that derangement in folate metabolism and the relevant status of apo-HC, apo-TC, and tHcy might be involved in the complex pathogenesis of NTDs.

Several previous studies had focused on the polymorphisms on the gene of *MTR* and *MTRR* in different populations. The abnormal activity of *MTR* could lead to accumulation of methyl THF which could not be used in folate-dependent reactions, such as thymidylate biosynthesis which is involved in NTD pathogenesis.^24^ It was corroborated that the polymorphism 2756A>G on *MTR* gene could convert asparthione to glycine.^25,26^ The subjects with the genotype GG were reported to have lower level of homocysteine in plasma compared with AA and AG in previous researches.^27–31^

The activation of *MTR* is maintained by *MTRR* which could demethylate cobalamin (II) to cobalamin (I) using *S*-adenosylmethionine (SAM). In has been proved that the SNP 66A>G on *MTRR* gene result in a conversion in the *MTRR* enzyme at position 22 by replacing isoleucine with methionine (I22M) and further deviate the ordinary function of *MTRR* which would express as decreasing the efficient repair of *MTR*.^16,32^ Most of the previous studies revealed that subjects with the GG genotype had degraded level of homocysteine compared with subjects with other genotypes.^33^

**FIGURE 3:**
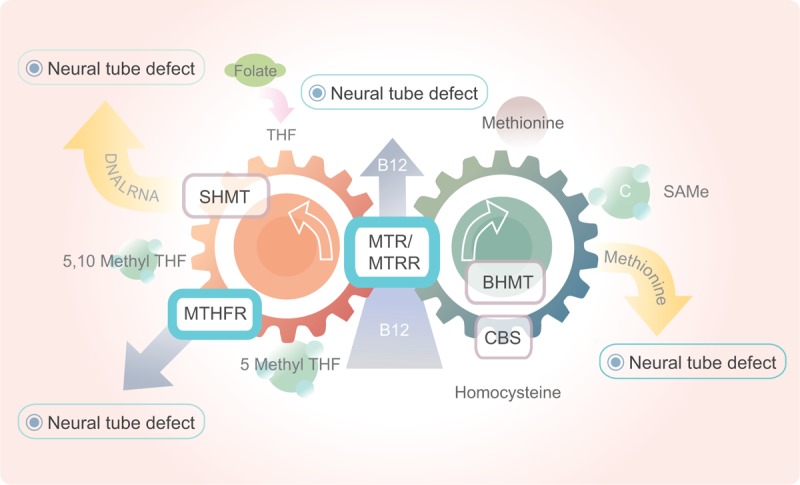
The process of folate cycle related to neural tube defect. BHM, betaine–homocysteine methyltransferase; CBS, cystathionine-β-synthase; *MTR*, methionine synthase; *MTRR*, methionine synthase reductase; MTHFR, methylene tetrahydrofolate reductase; RFC, the reduced folate carrier; SAH, *S*-adenosylhomocysteine; SAM, *S*-adenosylmethionine; SHMT, serine hydroxymethyltranferase; THF, tetrahydrofolate.

The SNPs we studied in the present study were not the only genetic factors of NTDs. The carbon atoms’ reduction at the oxidation levels of methyl, formyl, or methylene and the link to nitrogen were both involved in folate metabolism which would processes under a complicated pathway of many enzymes. To data, many folate pathway relevant SNPs have been investigated to the NTDs’ etiology, such as the SNPs on the gene of 5,10-methylenetetrahydrofolate reductase (MTHFR), serine hydroxymethyltranferase 1 (SHMT1), cystathionine-beta-synthase (CBS), and betaine–homocysteine methyltransferase (BHMT). The simplified version of the mechanisms of these SNPs is revealed in Figure 1. As we stated before, *MTR* gene could encodes the enzyme of vitamin B_12_-dependent and further convert 5-methyl THF and homocysteine to THF and methionine, respectively. Further, the genes above combine with the *MTR* and *MTRR* gene to finish the circle by the transfer from methyl group to homocysteine.^34,35^ As in the folate cycle, folate would convert in the form of tetrahydrofolate (THF) as the bioactive form firstly and further implement its function in folate metabolism (Figure 3). The conversion could be done under the function of the SHMT gene. Besides, MTHFR also performs a central role in the metabolism of folate by catalyzing the transfer of 5,10-methyleneTHF to 5-MTHF irreversibly and catalyzing the interconversion of serine and glycine. Likewise, CBS could catalyze the conversion of homocysteine to cysteine irreversibly. Consequently, the polymorphisms on these genes were supposed to disturb the regular function of relevant enzymes and lead to the suffering of NTDs. Similarly, the SNPs on BHMT gene could lead to the hyper of homocysteine and further result in NTDs.

Several limitations which could lead to inaccurate results have to be considered in our study. Firstly, the sample size of present study was relative small. Secondly, the outcome of NTDs varies among several symptoms which might not be considered severely and we did not restrict the NTDs type. Thirdly, environmental bias, like diet, smoking, and alcohol consumption, were not considered in the present study will also be a limitation. Certainly, gene–environment and gene–gene interaction also have influence on the present study.

In order to minimize the limitation of individual study with small sample size and the controversy of relevant studies, we performed a meta-analysis. According to our meta-analysis, *MTRR* 66A>G was significantly associated with the risk of NTDs widely which also elucidated that the mutation of folate related gene could lead to the suffering of NTDs. All the analysis was performed with no publication bias. Some studies with relative small sample size were also included in our meta-analysis, which also could be the limitation of the present analysis. The diversity of each study might be resulted from the bias of population or environmental stratification bias.

In conclusion, our result provide evidence that *MTRR* 66A>G and *MTR* 2756A>G were correlated with the increased risk of NTDs significantly. Moreover, the conclusion was supported by the further meta-analysis that *MTRR* 66A>G was associated with an increased NTDs risk. However, the other SNPs were not able to support the association significantly. Further study based on more detailed stratification and large populations were recommended in the research about folate relevant polymorphisms of NTDs.
